# Comparison between the efficacy of terbinafine and itraconazole orally vs. the combination of the two drugs in treating recalcitrant dermatophytosis

**DOI:** 10.1038/s41598-023-46361-z

**Published:** 2023-11-03

**Authors:** Zinab Rabia Abo Almaati Hassaan, Hassan Abou Khodair Mohamed, Ramadan Mohamed Eldahshan, Mohamed L. Elsaie

**Affiliations:** 1 Damietta Dermatology and Leprosy Hospital, Damietta, Egypt; 2 Department of Dermatology, Venereology and Andrology, Damietta Faculty of Medicine, Al-Azhar University, Damietta, Egypt; 3 Department of Dermatology, Venereology and Andrology, Medical Research and Clinical Studies Institute, National Research Centre, Giza, Egypt

**Keywords:** Medical research, Epidemiology

## Abstract

Fungal infections are a challenging to treat cutaneous condition. Approximately 20–25% of humans are affected by superficial fungal infections that invade and multiply within keratinized tissues. To compare the efficacy of either terbinafine or itraconazole orally versus the combination of the two drugs in the treatment of recalcitrant dermatophytosis. The current study included 45 patients with recalcitrant dermatophytosis who were distributed into 3 groups (each of 15 patients); Group A received terbinafine 250 mg twice a day for 4 weeks. Group B received itraconazole 200 mg twice a day for 4 weeks. Group C received terbinafine 250 mg once daily and itraconazole 200 mg once daily for 4 weeks. The patients were followed up for 12 weeks after initiation of treatment by clinical and microbiological assessment to determine the cure rate. At the end of twelve weeks, 12 (80%) patients in group A; 13 (86.7%) patients in group B and 15 (100%) patients in group C were completely cured. Despite of cure rates being higher in the combined group C; yet results were not statistically significant (p = 0.207). Clinical cure rates were non significantly higher in itraconazole + terbinafine combined group (p = 0.207). Combination of terbinafine and itraconazole had a higher clinical and mycological cure rate when compared to the use of either drug alone as monotherapy. Further randomized, multicenter, large cohort studies are warranted to validate the use of combination antifungal treatments.

## Introduction

Fungal infections are a challenging to treat common cutaneous condition. Approximately 20–25% of humans are affected by superficial fungal infections which can invade and multiply within keratinized tissues^1^.

Systemic therapy for dermatophytosis is indicated for extensive, recurrent and non responsive cases. Moreover; skin infections involving multiple sites and non responsive to topical treatment require using systemic antifungals. Wide variety of systemic antifungals is available for the treatment of tinea which consists of terbinafine, griseofulvin, itraconazole and fluconazole. Out of these, the commonly prescribed drugs are itraconazole and terbinafine as the other two require a longer duration^2^.

Owing to its mycological properties, terbinafine acting by inhibiting the enzyme squalene epoxidase was considered a first line treatment of choice when used in daily doses of 250 mg for two weeks and achieved cure rates of up to 90%. In recent years; terbinafine resistance emerged from overuse of the drug and resulted in increased clinical failures and relapses^3^.

On the other hand, itracanozole used at daily doses of 100 mg or 200 mgs for two weeks or one week has shown good results in treating dermatophytosis by inhibiting ergosterol synthesis^4^.

In recent years, there had been an increased resistance to systemic antifungals. And there had been a quest for finding an effective treatment with minimal relapse rates. A combination therapy of systemic antifungal drugs with different mechanism of action can enhance the cure rate and helps prevent drug resistance based on the concept of synergistic and additive effects of two or more drugs^5^.

Taking into consideration the lack of studies in literature on combination treatments, we aimed to compare the efficacy of either terbinafine or itraconazole orally versus the combination of the two drugs in the treatment of recalcitrant dermatophytosis.

## Patients and methods

This was a prospective randomized observational study that was carried out at the outpatient clinic of dermatology and venerology department's outpatient clinic et al.-Azhar University Hospital in New Damietta, Egypt. The study was conducted over nine months duration, from January 2023 till August 2023.

### Sample size

Sample size calculation was based on cure rate between different treatment lines of recalcitrant dermatophytosis using single or combined treatment retrieved from previous research by Singh et al., 2020 Using G*power version 3.0.10 to calculate sample size based on difference of 55.8%, 2-tailed test, α error = 0.05 and power of 80.0%; a sample size of 12 patients was determined sufficient for each group.

The enrolled patients were randomly categorized into three groups, A, B and C, each with 15 patients. Group a (ɳ = 15) received terbinafine 250 mg twice a day for 4 weeks. Group b (ɳ = 15) received itraconazole 200 mg twice a day for 4 weeks while group c (ɳ = 15) received terbinafine 250 mg once daily and itraconazole 200 mg once daily for 4 weeks. Randomization was done via a computer-generated random number sequence using the MS Excel software and one of the author assigned participants to the interventions.

Subjects were included if they fulfilled recalcitrance of dermatophyte infection based on the following criteria; complaining of cutaneous dermatophytosis for more than 6 months to 1 year with or without recurrence in spite of being adequately treated; those with two or more episodes of dermatophyte infections in the previous 12 months; recurrence of lesions within few weeks (< 6 weeks) after completion of the treatment and adults with relapsing dermatophytes after an infection-free interval of (6–8 weeks) following clinical cure.

The cases with the following criteria were excluded; immuno-compromised patients, patients with known active liver, cardiac, renal, or neurological diseases, patients with uncontrolled diabetes mellitus, a history of hypersensitivity to terbinafine or itraconazole, pregnant or lactating females, patients who had received oral antifungal medication or topical antifungal treatment during the previous 4 weeks and any patient with secondary bacterial infections on top of the dermatophytic lesion.

The study gained approval from the local ethical committee of the faculty of medicine, Al-Azhar University (DFM-IRB00012367-22-12-001). All patients felt free to withdraw from the study at any time point, based on their request. Patient confidentiality was preserved, and the collected data were used only for scientific purposes.

The cases were subjected to the following: history taking (including demographic data, analysis of the current condition and associated medical or surgical data) and clinical examination to determine the lesions extension, associated symptoms and to exclude any systemic diseases.

### Clinical and mycological assessments

Various clinical signs and symptoms (scaling, pruritis, erythema) were rated according to a four-point scale from 0 to 3 (0 = absent, 1 = mild, 2 = moderate and 3 = severe). Patients were checked for residual changes or recurrence two weeks; four weeks (after treatment ended), eight weeks, and twelve weeks after treatment had begun.

### Sample collection

The diagnosis was made by a clinical examination and then confirmed by a microbiological analysis. Direct microscopy with a 10% potassium hydroxide (KOH) mount was used for analyzing skin scrapings. Each smear was examined in detail at 400 × magnification at enrollment and at the end of the fourth week. A full blood picture was taken in addition to liver and renal function tests.

### Follow up

A clinical evaluation was performed during each visit. Patients were monitored for at least 12 weeks post treatment to look for signs of recurrent dermatophytosis. The therapy's effectiveness was assessed every four weeks. When there were no visible symptoms (such as scaling, erythema, and pruritus) and a negative KOH, patients were deemed cured.

At each visit, photographic pictures were collected and compared to prior images to determine clinical response; Treatment failure was defined as a lack of significant (> 50%) clinical improvement after 4 weeks of treatment or the emergence of new lesions/extension of old lesions at any time throughout treatment. Treatment efficacy was defined by complete cure that includes both clinical cure (clinically completely normal skin) and mycological cure (negative KOH microscopy).

### Statistical analysis of data

The data collected were coded, processed and analyzed with SPSS version 27 for Windows® (Statistical Package for Social Sciences) (IBM, SPSS Inc, Chicago, IL, USA). Qualitative data as number (frequency) and percent was presented. The Chi-Square test (or Monte-Carlo test) made the comparison between groups.

The Kolmogorov–Smirnov test tested quantitative data for normality. Parametric data were expressed as median ± SD while the non-parametric data were expressed as median (Range). To compare three groups with normally distributed quantitative variables, one-way analysis of the variance (One-way ANOVA) test was used and Kruskal–Wallis test was used if the data were abnormally distributed. Stuart Maxwell testwas used to compare between two dependent groups with qualitative variable (of more than two levels). For all tests, P values < 0.05 are considered significant.

### Ethics approval

This study protocol was reviewed and approved by ethics committee on human research by Al Azhar Damietta faculty of medicine (DFM-IRB 00012367-22-12-001). All methods were performed in accordance with relevant guidelines and regulations.

### Consent to participate

Written informed consents were received from participants upon explanation of the study.

## Results

60 cases were assessed for eligibility; 45 of which were eligible for the study and 15 were excluded for not fulfilling inclusion criteria. The enrolled patients were randomly categorized into three groups, A, B and C, each with fifteen patients. The patient characteristics were comparable in all the groups. The mean age of the studied groups (A, B and C) was 41.53 ± 15.73, 31.87 ± 13.34 and 39.0 ± 14.19 respectively. Statistically, 82.2% of the overall participants were females. No significant difference was detected regarding gender, occupation, co morbid conditions or family history of dermatophytosis among all groups (P = 0.516) (Table 1).

**Table 1:** Socio-demographic characteristics and clinical diagnosis of the studied groups.

	Group A n = 15	Group B n = 15	Group C n = 15	Test of significance
Age/years Mean ± SD	41.53 ± 15.73	31.87 ± 13.34	39.0 ± 14.19	F = 1.80 P = 0.177
Sex				MC = 3.95 P = 0.139
Male	5(33.3)	1(6.7)	2(13.3)	
Female	10(66.7)	14(93.3)	13(86.7)	
Occupation				
Not working	1(6.7)	0	1(6.7)	MC = 10.29 P = 0.113
Manual worker	4(26.7)	1(6.7)	1(6.7)	
Students	0	6(40)	3(20)	
Housewives	10(66.7)	8(53.3)	10(66.7)	
Co-morbidities	4(26.7)	7(46.7)	6(40.0)	χ^2^ = 1.32 p = 0.516
Family history (+ ve)	5(33.3)	4(26.7)	8(53.3)	χ^2^ = 2.46 p = 0.293
Diagnosis				
Tinea cruris	10(66.7)	11(73.3)	14(93.3)	MC = 3.34, p = 0.188
Tinea corporis	9(60)	6(40)	4(26.7)	MC = 3.46, p = 0.177

F: One Way ANOVA test, MC: Monte Carlo test, χ^2^ = Chi-square test.

The most common clinical variant was tinea corporis and cruris. Among group a; 66.7% were diagnosed as tinea cruris and 60% as tinea corporis. For group B; 73.3% tinea cruris and 40% tinea corporis while in group C; 93.3% were tinea cruris and 26.7% tinea corporis. No significant difference among case distribution was noticed in all groups (p = 0.188; p = 0.177) (Table 2).

**Table 2:** Comparison of recurrence rate, KOH results and cure rate between groups.

Variables	Group A n = 15(%)	Group B n = 15(%)	Group C n = 15(%)	Test of significance
Recurrence rate				
4 weeks	1(6.7)	1(6.7)	2(13.3)	MC = 0.549 p = 0.760
8 weeks	2(13.3)	2(13.3)	2(13.3)	p = 1.0
12 weeks	6(40)	4(26.7)	1(6.7)	p = 0.102
KOH results				
Baseline KOH	15(100)	15(100)	15(100)	p = 1.0
KOH after treatment	3(20.0)	2(13.3)	0	p = 0.207
Cure rate				
Cured	12(80)	13(86.7)	15(100)	MC = 3.15 P = 0.207
Failed	3(20)	2(13.3)	0	

*MC test* Monte Carlo test, *KOH* potassium hydroxide.

At the end of twelve weeks, 12 (80%) patients in group A; 13 (86.7%) patients in group B and 15 (100%) patients in group C were completely cured. Despite of cure rates being higher in the combined group C; yet results were not statistically significant (p = 0.207). Clinical cure rates were non significantly higher in itraconazole + terbinafine combined group (p = 0.207). GIT upset was the most frequent adverse event reported by almost all patients in all groups. Other side effects were minimal and non significant among all groups (Table 3).

**Table 3:** Comparison of side effects between studied groups.

Side effects	Group A n = 15 (%)	Group B n = 15 (%)	Group C n = 15 (%)	Test of significance
GIT upset	14 (93.3)	15 (100)	15 (100)	MC = 2.05, P = 0.360
Headache	1 (6.7)	0	0	MC = 2.05, P = 0.360
Elevated liver enzymes	2 (13.3)	3 (20)	2 (13.3)	MC = 0.338, P = 0.844
Hypersensitivity	1 (6.7)	0	0	MC = 2.05, P = 0.360
Loin pain	0	3 (20)	4 (26.7)	MC = 4.39, P = 0.111

*MC* Monte Carlo test; *GIT* gastro intestinal tract.

**Figure 1:**
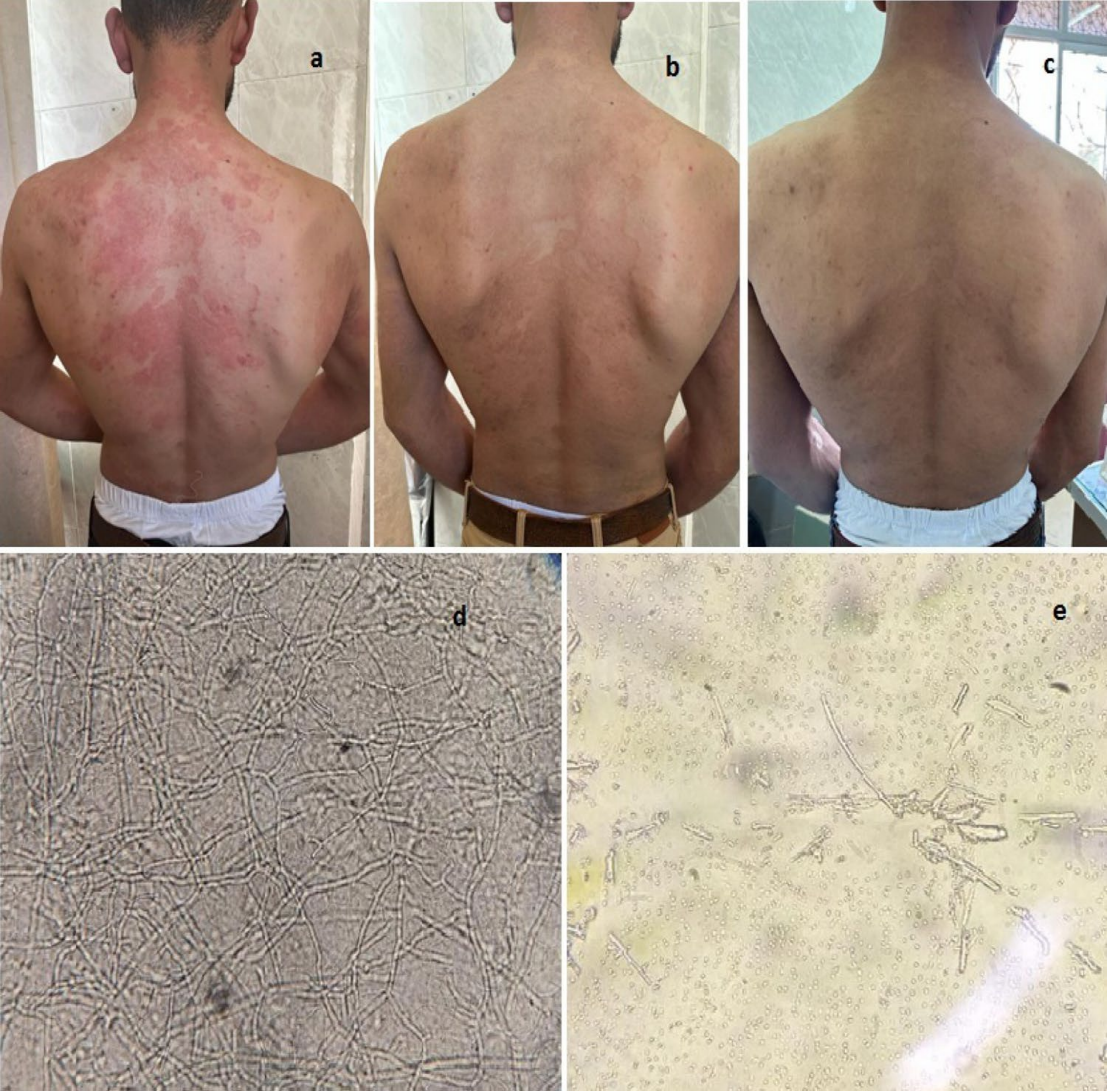
Clinical image of a twenty five years old male complaining of (**a**) recalcitrant tinea corporis of the back before treatment, (**b**) two weeks after treatment and (**c**) four weeks after treatment (**d**) Positive microscopic examination using koh showing (large amount of branching fungal hyphae) and (**e**) Negative microscopic examination using KOH (absent fungal hyphae) four weeks after treatment.

**Figure 2:**
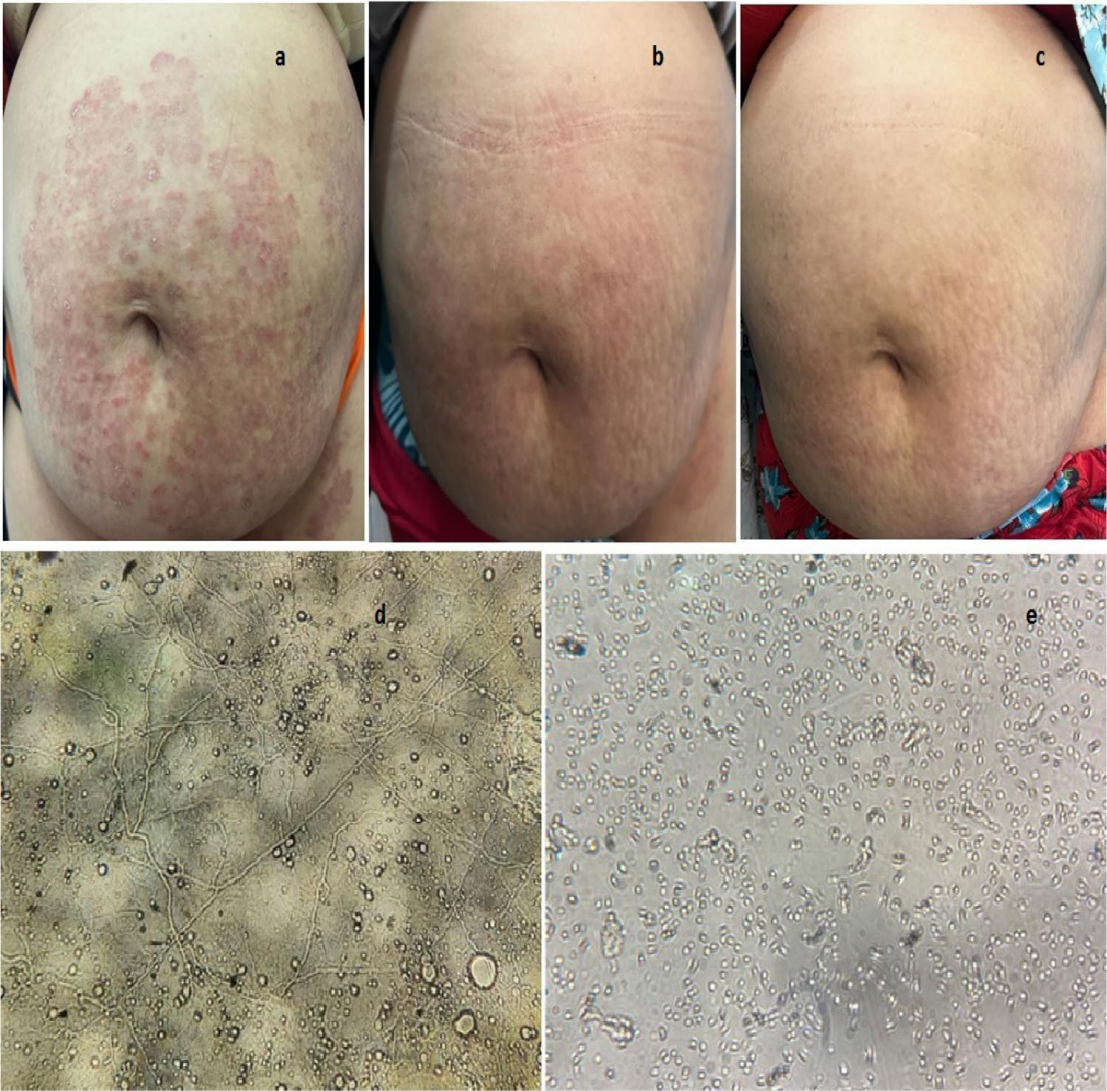
Clinical image of a forty years old female complaining of (**a**) recalcitrant tinea corporis of the abdomen before treatment, (**b**) two weeks after treatment and (**c**) four weeks after treatment (**d**) Positive microscopic examination using koh showing (large amount of branching fungal hyphae) and (**e**) Negative microscopic examination using KOH (absent fungal hyphae) four weeks after treatment.

Recurrence rates assessed at 4, 8 and 12 weeks respectively showed no significance among all groups (p = 0.760, p = 1, p = 0.102 respectively). Following treatment, scaling, pruritis and erythema scores were significantly improved as compared to the baseline values in all groups (p < 0.03, p < 0.001, p < 0.001 respectively), slightly higher non significant improvement was noted in group C. This result indirectly implies that the clinical features of patients at the end of treatment improved in all groups due to the treatment given (Table 4; Figs. 1, 2).

**Table 4:** Comparison between erythema, scaling and pruritis scores at baseline and after treatment.

		Group A n = 15(%)	Group B n = 15(%)	Group C n = 15(%)	Test of significance
Scaling	Before treatment				MC = 6.88 p = 0.142
	Mild	7(46.7)	2(13.3)	2(13.3)	
	Moderate	5(33.3)	8(53.3)	6(40)	
	Severe	3(20)	5(33.3)	7(46.7)	
	After treatment				MC = 2.56 P = 0.634
	No	3(20)	7(46.7)	5(33.3)	
	Mild	10(66.7)	7(46.7)	8(53.3)	
	Moderate	2(13.3)	1(6.7)	2(13.3)	
P value		0.003*	0.001*	0.001*	
Pruritis	Before treatment				MC = 7.94 P = 0.091
	Mild	4(26.7)	0	1(6.7)	
	Moderate	5(33.3)	10(66.7)	6(40)	
	Severe	6(40)	5(33.3)	8(53.3)	
	After treatment				MC = 4.78 p = 0.309
	No	5(33.3)	8(53.3)	10(66.7)	
	Mild	9(60)	7(46.7)	5(33.3)	
	Moderate	1(6.7)	0	0	
P value		0.002*	0.001*	< 0.001*	
Erythema	Before treatment				MC = 8.49 P = 0.075
	Mild	3(20)	0	1(6.7)	
	Moderate	5(33.3)	10(66.7)	4(26.7)	
	Severe	7(46.7)	5(33.3)	10(66.7)	
	After treatment				MC = 2.92 P = 0.233
	No	6(40)	10(66.7)	10(66.7)	
	Mild	9(60)	5(33.3)	5(33.3)	
P value		0.001*	0.001*	0.001*	

Used test: Monte Carlo test, Stuart Maxwell test.

## Discussion

In this study, combination of terbinafine with itraconazole produced non significantly higher clinical cure rates (p = 0.207) as compared to itraconazole and terbinafine monotherapy. Despite of cure rates being higher in the combined group C; yet results were not statistically significant (p = 0.207).

In the current study, the mean age of the studied groups was 41.53 ± 15.73, 31.87 ± 13.34 and 39.0 ± 14.19 for groups A, B & C, respectively. The majority of the patients were females (82.2%). This is was in accordance with the study by Ramesh et al.^6^ who reported a female prevalence of (64.9%). In other studies, by Singh et al.^7^ and Sharma et al.^8^ male predominance was reported to be (74.1% and 70%; respectively). The difference in gender differences could be to the nature of the study population, cultural differences or sample size variance; yet female prevalence could be related to the cosmetic concern, impact or stigma of fungal conditions on female population.

In the past, terbinafine, in the dosage of 250 mg/day, has shown consistent efficacy against dermatophytosis, achieving more than 90% cure rates at a dose of 250 mg/day when admonstered^9^. Recently resistance to terbinafine treatment in the previously mentioned doses has led some physicians to increase doses or provide combination therapies^10^.

In this study, itraconazole and terbinafine achieved comparable cure rates when used alone and achieved less non significant cure rates to the itraconazole + terbinafine combination. This was comparable to Shah et al. who reported significant comparable cure rates of itraconazole and terbinafine when used alone^11^. Singh et al. reported itraconazole to be more effective when compared to terbinafine^7^.

Majid et al. achieved only 43% cure rate after two weeks of daily 250 mg terbinafine oral treatment in dermatophytosis^12^. Other studies showed the mycological cure rate of terbinafine as 74% and 71% respectively^13,14^. In another study, three-week therapy of terbinafine 250 mg/day showed a 35% cure rate, compared to 50% cure rate using itraconazole 200 mg/day^8^.

The practice of combination therapy has not been widely studied and published despite of being much practiced in many medical settings. The current study showed a comparatively better complete clinical cure rate with terbinafine and itraconazole (100%). This value was much higher compared to the study by Ramesh et al.^6^ that reported 48.3% complete cure and more comparable to the studies conducted by Singh et al. and Sharma P et al., that reported 79.2% and 90% clinical and mycological cure rate after three weeks and four weeks of combination therapy respectively^7,8^.

Other studies assessed combination therapies for recurrent fungal infections^15,16^. One study demonstrated that terbinafine with sertaconazole achieved a better but non significant efficacy than itraconazole and sertaconazole combination. Successful complete response without recurrence was obtained following combination of oral (20 mg/day) and itraconazole (200 mg/day) along with daily application of topical sertaconazole^15^. Another recent study reported a significant clinical cure in 70% of the (itraconazole + isotretinoin combination) treated recurrent tinea patients when compared to only cure in 56.7% of itraconazole only treated patients^16^.

The most common side effects with terbinafine are gastric upset, headache, altered taste, altered liver function tests and rash; rarely, it can cause blood dyscrasias and hepatitis. Itraconazole can cause gastric upset, headache, taste alteration, and jaundice, and rarely, it can cause hypokalemia, torsades de pointes, and heart failure. In the current study, gastric upset was the most commonly reported adverse event, while hypersensitivity, loin pain and liver enzymes were unremarkably and non significantly reported among 3 patients in all groups.

The study is limited by its small sample size, female predominance. Mycological culture was not done and follow up durations were short. Moreover; the use of fixed doses represented a limitation. Another limitation was not investigating minimum inhibitory concentration of the combination of the two drugs.

## Conclusion

In conclusion combination of the drugs terbinafine and itraconazole had a higher clinical and mycological cure rate when compared to the use of either drug alone as monotherapy. It is possible that the lack of a statistically significant difference is attributable to the small sample size of the cases that were included. Further randomized, multicenter, large cohort studies using in vitro characterization and investigating the minimal inhibitor concentrations of combined antifungal treatment are warranted to validate the use of combination antifungal treatments and establish optimal safe dosage in the management of recalcitrant dermatophytosis.

## Data Availability

The data that support the findings of this study are available from the corresponding author upon reasonable request.

## Abbreviation

KOH: Potassium hydroxide

## Code availability

All authors confirm all data and materials as well as software applications or custom code support their published claims and comply with field materials.
